# A Validated Mass Spectrometry Platform for Oxysterol Analysis of Single Human Gastruloids and Liver Organoids

**DOI:** 10.1021/acs.analchem.5c07140

**Published:** 2026-02-24

**Authors:** Kristina Sæterdal Kømurcu, Malgorzata Elzbieta Zawadzka, Igor Meszka, Aleksandra Aizenshtadt, Helena Hrušková, Lydia Emilie Aakervik, James L. Thorne, Steven Ray Wilson, Stefan Johannes Karl Krauss, Hanne Røberg-Larsen

**Affiliations:** † Department of Chemistry, 87361 University of Oslo, P.O. Box 1033 Blindern, Oslo 0315, Norway; ‡ Hybrid Technology Hub - Centre of Excellence, Institute of Basic Medical Sciences, 6305 University of Oslo, P.O. Box 1110 Blindern, Oslo 0317, Norway; § School of Food Science and Nutrition, 4468 University of Leeds, Leeds LS2 9JT, United Kingdom; ∥ Department of Immunology and Transfusion Medicine, Oslo University Hospital, Oslo 0424, Norway

## Abstract

Oxysterols, i.e., hydroxylated cholesterol metabolites, are associated with various signaling pathways and diseases. Their low abundance and structural complexity create analytical challenges, particularly in small sample sizes. We here present an optimized and validated miniaturized sample preparation method that enables oxysterol detection and quantification in single stem cell-derived 3D cell aggregates, as exemplified in human liver organoids (stem cell-based 3D liver models) and human gastruloids (stem cell-based embryo models) using liquid chromatography–mass spectrometry (LC–MS). The method, utilizing enzyme-assisted derivatization with Girard-T reagent, allowed a 10-fold decrease in starting material compared to conventional methodology while maintaining sensitivity and precision. A validation based on Eurachem guidelines confirmed quantitative performance and reproducibility across days and operators. In addition, we introduce a tailored normalization method, allowing same-sample measurements of oxysterols and the total protein content. The miniaturized method enabled successful detection and quantification of oxysterols of expected presence (e.g., 26-hydroxycholesterol), as well as unexpected (24S-hydroxycholesterol) and unknown oxysterols. Using our updated method, we could reveal significant heterogeneity among individual organoids and gastruloids, both between and within cell sources/protocols. Overall, we provide a reliable and high-sensitivity method for analyzing oxysterols in limited biological samples, opening opportunities for further insights into their roles in, e.g., liver function and early embryogenesis.

## Graphical abstract

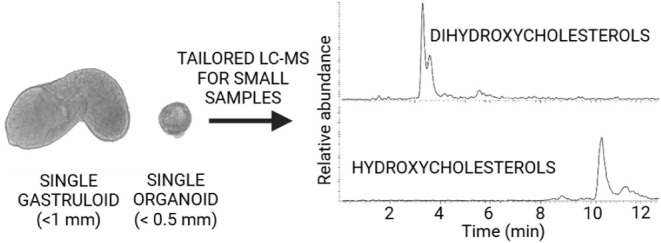

## Introduction

Oxysterols are transport forms of cholesterol, formed either enzymatically by CYP P450 enzymes or through autoxidation.^1^ These biologically active metabolites serve as intermediates in bile acid synthesis in the liver and play key roles in multiple biological processes, e.g., as ligands for nuclear receptors such as liver X receptors (LXRs) and estrogen receptors,^2–4^ as transporters of cholesterol across the blood–brain–barrier,^5^ and as modulators of the developmental Wnt and Hedgehog signaling pathways.^6–8^

Due to their bioactivity, oxysterols have been implicated in a wide range of diseases,^9^ including metabolic dysfunction-associated steatotic liver disease (MASLD),^10^ a variety of cancers such as breast^11,12^ and prostate cancer,^13,14^ atherosclerosis,^15^ diabetes mellitus,^16^ and Alzheimer’s disease.^17–19^ In addition, oxysterols are established biomarkers for the inborn disorders of cholesterol biosynthesis Smith–Lemli–Opitz disease,^20^ where accumulation of sterol precursors and oxysterols accompanies multiple congenital malformations, and Niemann–Pick disease.^21^

Oxysterols are notoriously challenging metabolites to quantify for several reasons. The isomeric nature of many key oxysterols demands highly efficient chromatographic separation. Traditional separation approaches such as gas chromatography (GC) demand derivatization, often including a hydrolysis step.^22^ However, hydrolysis-including steps do not allow the distinguishing between free biologically active oxysterols and esterified oxysterols. With liquid chromatography, a hydrolysis step is typically avoided, allowing free oxysterols to be measured independently. However, the often-neutral oxysterol structure is generally difficult to ionize using electrospray ionization (ESI), the main interface for liquid chromatography–mass spectrometry (LC–MS).^23^ Although methods exist for native oxysterol analysis with LC–ESI-MS,^24^ these methods are not suited for high-sensitivity analysis, as is often the case with GC–MS approaches.^25^ Moreover, native analysis of oxysterols is associated with nonspecific and multiple fragmentation patterns in MS/MS.^23^ Other approaches such as APPI and APCI may allow for increased ionization^26–29^ but also result in nonspecific fragmentation in MS/MS. Hence, today’s “go-to” approach for oxysterol determination is therefore combinations of derivatization and LC–ESI-MS/MS which allow for sensitive and specific analysis.^25^

One of the established methods to improve detection sensitivity and specificity is the derivatization of oxysterols into hydrazones using Girard (P or T) reagent.^30^ In these approaches, cholesterol oxidase is first used to selectively oxidize the 3β-hydroxy group on the oxysterol backbone to a keto group, giving a 3-oxo-4-ene sterol. Further, the Girard reagent (T or P) reacts with the keto group to form a Girard hydrazone. This reaction introduces a permanent positive charge via the quaternary ammonium group in the Girard reagent, which significantly enhances ionization efficiency in positive-mode (ESI),^30^ in addition to providing structurally informative MS/MS spectra.^23^ The reaction steps take place at 37 °C and room temperature, reducing the risk of autoxidation. However, established Girard “charge-tagging” methods utilize high volumes of reagents for optimal reaction conditions, resulting in a high degree of dilution of samples, regardless of the sample’s starting amount. Thus, final derivatized sample volumes are in the range of 700–1000 μL, which is not beneficial when working with limited sample sizes.

Other methods, such as derivatization by picoline esters,^31,32^ provide high-sensitivity analysis of small sample sizes without extensive dilution. However, these reactions often include higher reaction temperatures, which could give higher chances of autoxidation.

Due to the challenges in detection and quantification, oxysterols are understudied relative to their importance in human health, and methods for their analysis in emerging tools and models of disease processes and drug effects on the human body are also lacking. Given that oxysterols have relevant biological functions related to disease and developmental processes, the ability to study them under sample-limiting conditions is crucial, particularly when using advanced model systems such as organoids and gastruloids.

Organoids and gastruloids are 3D cell structures grown *in vitro* from stem cells,^33,34^ mimicking key structural and functional characteristics of actual organs and embryogenesis,^35^ respectively. Organoids can be derived from human cells, creating models of physiology and metabolism that can increase efficiency and relevance for human-related studies compared to animal models.^36–38^

For example, liver organoids can be used as models for human-specific liver conditions such as MASLD,^36,37^ and in our previous work, we found an upregulation of 26-HC in MASLD-induced liver organoids.^38^ Gastruloids feature developing germ layers (mesoderm, endoderm, and ectoderm) and an emerging posterior–anterior (head–tail) axis.^35,39^ These simplified embryonic models, which can also feature organ-like structures, e.g., a beating cardiac-like domain, offer unique opportunities to investigate complex early developmental processes.^40^ Gastruloids are studied with a plethora of cell biological approaches, but less research has been performed with regard to lipid analysis and specific lipid classes. As oxysterols are known to be key modulators of the Hedgehog signaling pathway and Wnt/β-catenin signaling^41,42^ both being essential for human embryonic development, assessing their presence in stem cell-based embryo models such as gastruloids can provide valuable insights into the underlying mechanisms of early developmental processes.

One well-described challenge with liver organoids, but in particular with gastruloids, is their biological variance within an experimental setting and between experiments. Both liver organoids and gastruloids can vary significantly, e.g., in size, cellular composition, and growth dynamics, even when derived from the same source and cultured under identical conditions.^43,44^ Analyzing individual specimens allows for precise description of each specimen, leading to more accurate data interpretation. Hence, the study of single specimens has become increasingly important for understanding differences that might otherwise be masked in bulk analyses.^43–46^ Single specimen approaches enable high-resolution mapping of cellular diversity, lineage relationships, and functional responses within individual specimens, providing insights into both normal physiology/development and disease modeling.^46,47^ It is important to note that hitherto single specimen studies focus primarily on transcriptomics and RNA sequencing, while metabolomics/lipidomics and proteomics are investigated to a much lesser extent. These studies emphasize that analyzing single specimens is essential for understanding their biological variability.^48,49^ Nonetheless, to enable accurate comparison between single specimens, results need to be normalized, e.g., against total protein content, to account for differences in organoid size, cellularity, and overall biomass.^48,49^

In our study of sterols in single organoids and gastruloids, we developed a downscaled derivatization method enabling oxysterol quantification. Key goals were to ensure adequate identification and quantification of the analytes, map oxysterol presence in the samples, and assess the heterogeneity between individual liver organoids and gastruloids.

## Experimental Section

### Generation of Human Pluripotent Stem Cell-Derived Liver Organoids (hscLO)

Human pluripotent stem cell-derived liver organoids from human embryonic stem cells (hESC) (H1 cell line, Coriell Institute for Medical Research) and human induced pluripotent stem cells (hiPSC) (WTC-11 cell line, Coriell Institute for Medical Research) were generated using an established protocol, as published previously.^38,50,51^

### Human Gastruloids

The generation of human gastruloids was based on previously published protocols,^35,52,53^ with modifications (Meszka, Krauss et al., manuscript in preparation). Gastruloids were generated by aggregating human embryonic stem cells into uniform spheroids under low-attachment culture conditions, preceded by controlled exposure to defined morphogen signals. The aggregates were then maintained in fresh differentiation medium over 5 days. These conditions enable the emergence of polarized, elongated structures that display aspects of early axial patterning and germ-layer organization. All gastruloids were prepared following the same protocol from the same cell lines but in several batches (seeding 600 cells per well to generate one gastruloid and collection at day 5 after aggregation). All experiments involving human pluripotent stem cells and derived gastruloids were approved by the Regional Committees for Medical and Health Research Ethics (REK, Norway, Approval no. 522684; Project title: Supervised morphogenesis in gastruloids).

### Chemicals and Solutions

All reagents were HPLC- or MS-grade. Methanol (MeOH), acetonitrile (MeCN), isopropanol (*i*PrOH), and water were purchased from VWR. Glacial acetic acid (AcOH), formic acid (HCO_2_H), cholesterol oxidase, phosphate buffer, Girard’s reagent T, cholesterol-25,26,27-^13^C and 25-hydroxycholesterol (25-HC) were purchased from Sigma-Aldrich. 7β26-diHC, 7α26-diHC, 7β25-diHC, 7α25-diHC, 7α24S-diHC, 24S-HC, 26-HC, 7β26- diHC-*d*_6_, 7α25-diHC-*d*_6_, 25-HC-*d*_6_, and 26-HC-*d*_6_ were purchased from Avanti Polar Lipids. All stock solutions of standards and internal standards were prepared in *i*PrOH and stored at −20 °C. Working solutions of 200 pM standard mixture 1 (7β,25-diHC, 7α,25-diHC, and 7α,24S-diHC) and mixture 2 (7α,26-diHC, 7β,26-diHC, 22R-HC, 25-HC, 24S-HC, and 26-HC) in *i*PrOH were prepared from stock solutions. A combined mixture of 3 nM internal standard (7α,25-diHC-*d*_6_, 7β,26-diHC-*d*_6_, 25-HC-*d*_7_, and 26-HC-*d*_7_, IS) and 431 nM cholesterol-25,26,27^13^C (for autoxidation monitoring, AOM) in *i*PrOH was prepared from stock solutions. Solutions of 1.2 and 0.2 mg/mL cholesterol oxidase in 50 mM phosphate buffer (pH 7) were stored in aliquots at −80 °C, and fresh buffer was thawed before each use. Urea and ammonium bicarbonate (ABC) were purchased from Sigma-Aldrich. Buffer solution containing 6 M urea in 100 mM ammonium bicarbonate was freshly prepared before each analysis. Pierce BCA Assay Kit (Thermo Fisher Scientific) was used for protein determination. Kit’s protocol was followed for both calibration solution preparation and analysis with a Thermo Scientific Multiskan EX plate reader. Gel electrophoresis experiments were performed with Invitrogen SDS-PAGE kits, Bolt Bis-Tris Plus Mini Protein Gels, 4–12%, 1.0 mm, WedgeWell format, and a Mini Gel Tank (Thermo Fisher Scientific).

### Optimization of Derivatization Using Design of Experiments

To optimize a downscaled sample preparation protocol, a fractional factorial design was implemented using Design Expert software (Stat-Ease Inc.). A half-fraction Central Composite Design (CCD) was used, with a rotatable alpha of 2, and five variables; *i*PrOH (μL), cholesterol oxidase solution (μL solution, hence changing the μg amount added), methanol (μL), glacial acetic acid (μL), and Girard-T reagent (Table 1). The design resulted in 26 noncenter points and 6 center points, totaling 32 samples. The ratio between the combined total peak area of 5 oxysterols (7α26-diHC, 7β26-diHC, 25-HC, 24S-HC, 26-HC) and 25-HC-*d*_6_ internal standard was used as a response.

**Table 1:** Overview of the 5 Factors Used for the Experimental Design, Including Units and Limits

	Factor	Unit	Low	High	–Alpha	+Alpha
**A**	Isopropanol	μL	3	7	1	9
**B**	Cholesterol oxidase	μL	3	7	1	9
**C**	Methanol	μL	60	120	30	150
**D**	Glacial acetic acid	μL	2	6	0	8
**E**	Girard-T	mg	3	7	1	9

For method optimization, 120 μL of standard mixture 2 was evaporated to dryness before various amounts of *i*PrOH were added (according to the design), followed by 20 μL of phosphate buffer. The assigned amount of a 1.2 μg/μL cholesterol oxidase in phosphate buffer solution was added (resulting in a final amount of cholesterol oxidase between 1.2 and 11 μg) before incubation at 37 °C for 1 h. Assigned amounts of methanol, glacial acetic acid, and Girard-T reagent were added to the samples before incubation overnight. Before analysis, 80 μL of the prepared sample was combined with 20 μL of a 1 nM internal standard solution (derivatized after the original procedure^54^ to correct for possible MS signal drift).

### Optimized Derivatization Procedure

The dried sample or calibration solution was redissolved in 7 μL of *i*PrOH. Oxysterols were oxidized by adding 24 μL of 0.2 μg/μL cholesterol oxidase in phosphate buffer, followed by incubation at 37 °C for 1 h. Next, for derivatization, 40 μL MeOH, 4 μL glacial acetic acid, and 7 mg Girard-T were added to each sample/standard, with subsequent incubation overnight in darkness (Figure 1).

**Figure 1:**
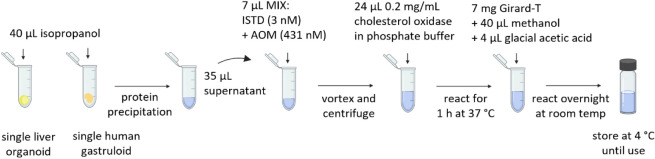
Overview of the sample preparation protocol for derivatization of oxysterols, allowing determination of oxysterols in single organoids and gastruloids. The sample preparation consists of the following steps: addition of internal standard and AOM mixture, mixing and centrifuging, addition of cholesterol oxidase followed by reacting for 1 h, addition of Girard-T, methanol, and glacial acetic acid, and derivatization overnight. Samples are stored at 4 °C until use.

### Calibration Solutions and Internal Standard Solutions

Calibration solutions in the concentration range of 25–500 pM analytes with 280 pM internal standard and 40 nM AOM were prepared by mixing appropriate volumes of 200 pM standard mixtures with 7 μL of IS/AOM mixture and evaporated to dryness to remove *i*PrOH before Girard-T derivatization, as described above. New calibration solutions were prepared for each assay.

### Liver Organoid and Gastruloid Samples

Samples were prepared by adding 40 μL of *i*PrOH for lipid extraction and protein precipitation to single organoids or gastruloids, before shaking and centrifugation at 4 °C for 15 min. Subsequently, 35 μL of the supernatant was transferred to a new vial containing 7 μL of the IS/AOM mixture. The sample was evaporated to dryness before Girard-T derivatization. The remaining pellet was used for protein measurements.

### Cell Medium

Cell medium samples (William’s E media, supplemented with 1% (v/v) Glutamax, 0.1% (v/v) insulin-transferrin-selenium, 0.1 μM dexamethasone, 0.5% (v/v) MEM nonessential amino acid solution, and 1% (v/v) knockout replacement for organoids and E6 media for gastruloids) were prepared by mixing 50 μL of neat media (not used in the cultivation of liver organoids or gastruloids) with 7 μL of the IS/AOM mixture. Samples were evaporated to dryness before Girard-T derivatization.

To test for the possibility of the presence of oxysterols with naturally occurring 3-keto groups in the samples, both liver organoids, gastruloids, and respective cell media were derivatized without the presence of cholesterol oxidase. In this case, the same procedure was used, except for adding 24 μL of pure phosphate buffer instead of the cholesterol oxidase solution. All samples and calibration solutions were stored at 4 °C prior to analysis.

### Precipitation and Protein Content Measurements

The remaining pellet after lipid extraction with *i*PrOH and removal of the supernatant for oxysterol derivatization was evaporated and redissolved in 7 μL of cold 6 M urea/100 mM ABC buffer. For protein measurements, the general procedure of the BCA kit was followed, with some modifications: 5 μL of sample, standards, blanks, or QCs were transferred to the 96-well plate and mixed with 100 μL of working reagent mixture (prepared according to the BCA kit procedure), before incubation for 2 h at 37 °C.

### SDS-PAGE Gel Electrophoresis

SDS-PAGE gel electrophoresis of the supernatant of protein pellets was conducted, following three different sample preparation procedures to assess the impact of *i*PrOH volume and sample buffer on the precipitation process. Lipids from one and five gastruloids were extracted with 40 μL of *i*PrOH, followed by evaporating to dryness both the pellet and the supernatant. Next, both were redissolved in 30 μL of water, and 10 μL of sample buffer was added accordingly. Such prepared samples were applied to the gel and processed according to the kit recommendation.

### Method Validation

The three oxysterols detected in organoid and gastruloid samples were included in the validation (24S-HC, 26-HC, and 7β26-diHC). The linearity, regression coefficient, intra- and interday analytical precision (given as relative standard deviation, RSD), repeatability between the operator, and relative error were validated. Six replicates of each sample were tested at three concentration levels: LLOQ = 50 pM, MLOQ = 200 pM, and HLOQ = 500 pM. The limit of detection (LOD) was calculated following Eurachem guidelines as 3 × *S*_0_′ (the standard deviation), based on >10 replicate measurements at low concentrations achieving <20% RSD. The limit of quantification (LOQ) was specified as 10 × *S*_0_′. The ratios for each peak were calculated by using the XCalibur software.

### LC–MS System

LC–MS conditions were as described in Kømurcu et al.^38^ with some smaller modifications. The online SPE column was a C18 column (Teknolab), and the loading mobile phase was delivered by a Hitachi L-7110 pump (Merck). The injection volume was 60 μL. The analytical column used was an ACE SuperPhenyl Hexyl (2.1 mm ID, 150 mm, 2.5 μm d_p_ core–shell particles) column from Advanced Chromatography Technologies LTD (Aberdeen, UK). An isocratic 7 min step of mobile phase composition 61/10/29 (v/v/v, H_2_O/MeOH/MeCN) at the start of separation enabled the separation of the dihydroxycholesterols before an increase in mobile phase strength to 56/10/34 (v/v/v, H_2_O/MeOH/MeCN) that was maintained for 5 min for the separation of hydroxycholesterols. A 2.5 min wash step followed using a mobile phase composition of 50/50 (v/v, B/C). Information about MS instrumentation, method parameters, and targeted analytes are found in Supporting Information (SI) SI-1 and Table S1. High-resolution MS information for gastruloid samples is included in SI-4.

### Calculation and Statistics

Design of Experiments (DoE) was conducted using Design Expert software (Stat-Ease Inc.). LC–MS data acquisition was performed with Chromeleon Xpress, and subsequent spectral analysis and quantification were carried out in Xcalibur (both software from Thermo Scientific). For statistical calculations, one-way ANOVA analysis, Grubbs test, and paired *t*-test were performed according to the Eurachem guidelines in Excel (Microsoft). The figures and graphs were made in BioRender App, PowerPoint, or Microsoft Excel.

## Results and Discussion

### Downscaled Sample Preparation

In our original sample preparation method^54^ for derivatization of oxysterols using Girard-T reagent (largely based on conventional methodology^55^), the final volume exceeds 700 μL. The method has previously been successfully applied to e.g., organoid media^38^ and cancer cell lines,^56^ cell line-conditioned media,^56^ and primary tumor samples.^54,57^ However, when working with more limited starting materials such as single organoids/gastruloids, the large final volume would contribute to a substantial dilution of the sample and hence a reduction in sensitivity. Hence, we aimed to reduce the final volume using a five-factorial Central Composite Design (CCD).^58^ Reaction conditions were optimized using a selection of hydroxycholesterols and dihydroxycholesterols: 25-HC, 24S-HC, and 26-HC, and the more hydrophilic 7β26-diHC and 7α26-diHC (Figure 2). Analyte amounts investigated were present in trace amounts (9 pg/sample), which is substantially lower than those used in previous sample preparation optimizations (up to 150 pg per sample).

**Figure 2:**
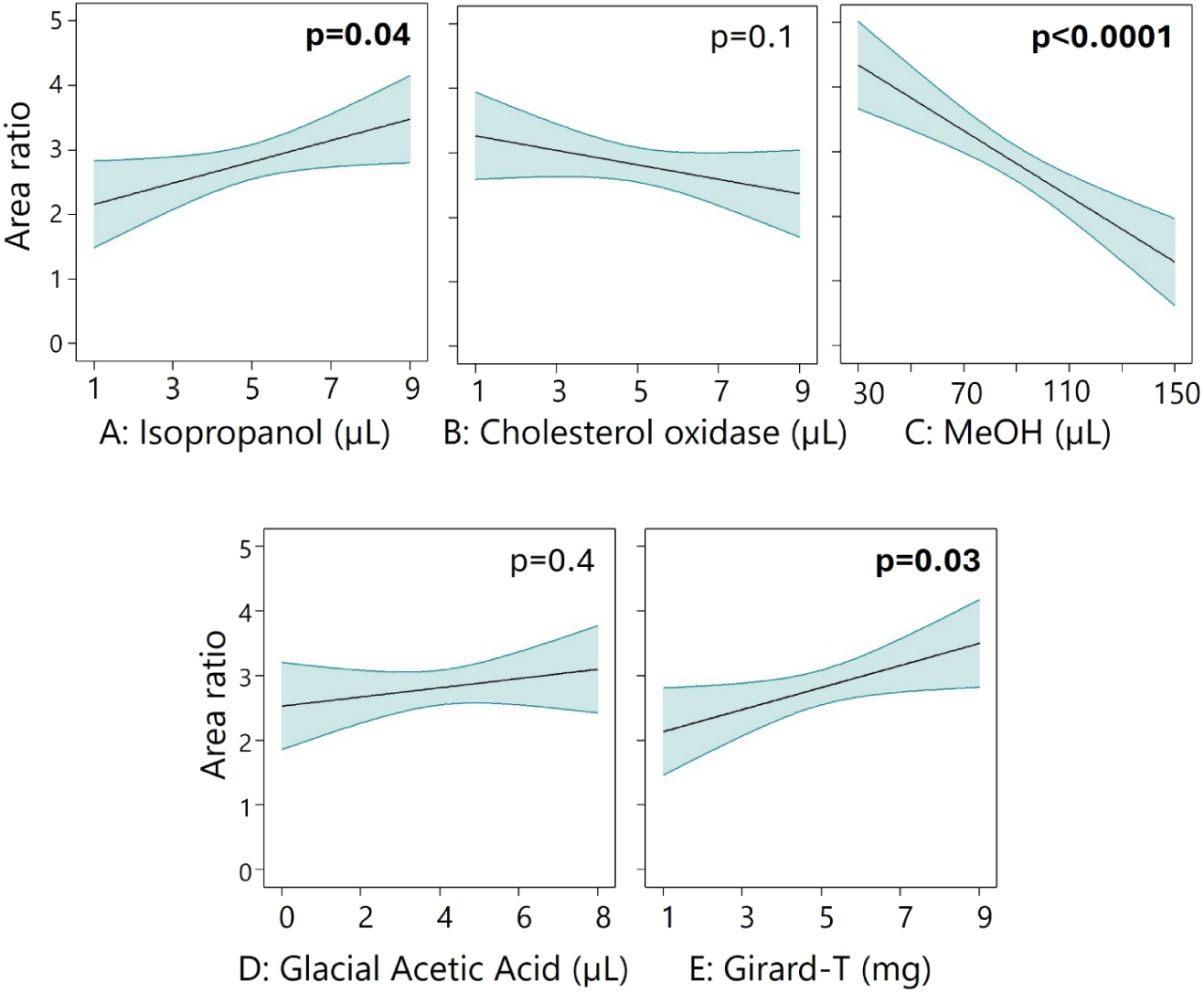
Area ratio effect of the amounts of the five reagents validated. Being dependent on each other, the other factors are set to center point values (5 μL, 5 μL,90 μL, 4 μL, and 5 μL for A–E respectively) when visualizing their effect individually. Significant *p*-values are written in bold.

Girard-tagging is dependent on the presence of methanol, and conventional methodology features a 70% methanol content (making up most of the final sample volume, i.e., 500 μL) to ensure efficient reactions. However, reducing the methanol volume (and hence the total alcohol percentage) was associated with a significantly increased signal (*p* < 0.0001, Figure 2C). A possible explanation is that trace amounts of oxysterols prepared overnight require less methanol than the larger oxysterol amounts prepared when the conventional methodology^55^ was developed. Manual inspection of the chromatograms confirmed an increased sensitivity with reduced methanol. Girard-T and *i*PrOH content significantly but modestly improve yield when used in higher amounts (*p* = 0.03–0.04). While early work suggested an *i*PrOH concentration of ∼10% to improve the reaction rate for the cholesterol oxidase enzyme,^59^ our CCD analysis suggested improved performance at somewhat higher levels (22%). The amount of cholesterol oxidase and glacial acetic acid did not significantly affect the sensitivity, although the complete absence of acid led to poor signal intensity, confirming its necessity.

The final volumes of the optimized downscaled method compared to our original method^54^ are found in Table 2. Although the CCD study indicated that lower volumes of methanol could be used, 40 μL was used for reproducible handling and to avoid solubility issues with the Girard reagents. Overall, this approach allowed us to reduce the final sample volume from 735 to 75 μL, resulting in a 10-fold reduction in sample dilution.

**Table 2:** Final Amounts in a New, Downscaled Sample Preparation Procedure, Based on the Experimental Design Results, Compared to Our Original Method.^54^

		Original Method		New Method	
Factor	Reagent	Amount	% of total volume	Amount	% of total volume
A	Isopropanol	20 μL	2.7	7 μL	9.3
B	Cholesterol oxidase	6 μg	-	4.9 μg	-
	Phosphate buffer	200 μL	27.2	24 μL	32.0
C	Methanol	500 μL	68.0	40 μL	53.3
D	Glacial acetic acid	15 μL	2.0	4 μL	5.3
E	Girard-T	15 mg	-	7 mg	-
	**Total amount**	735 μL		**75 μL**	

### Validation of the Optimized Sample Preparation Method

A method validation demonstrated that the optimized derivatization protocol and analytical workflow are robust and reliable for quantitative applications. The linearity across the tested concentration range (50–500 pM, *R* ^2^ > 0.99) indicates the method is well-suited for accurate quantification of analytes within this range; see Figure 3A for a representative chromatogram. Precision values were below 20% RSD for all analytes (and below 10% for most of them) for measurements performed on different days and by different operators (*n*_intra_ = 6, *n*_inter_ = 4), further confirming the reproducibility of the approach, see Figure 3B. This level of consistency underscores the method’s robustness and suggests minimal influence of external variables such as operator handling or day-to-day variability.

**Figure 3:**
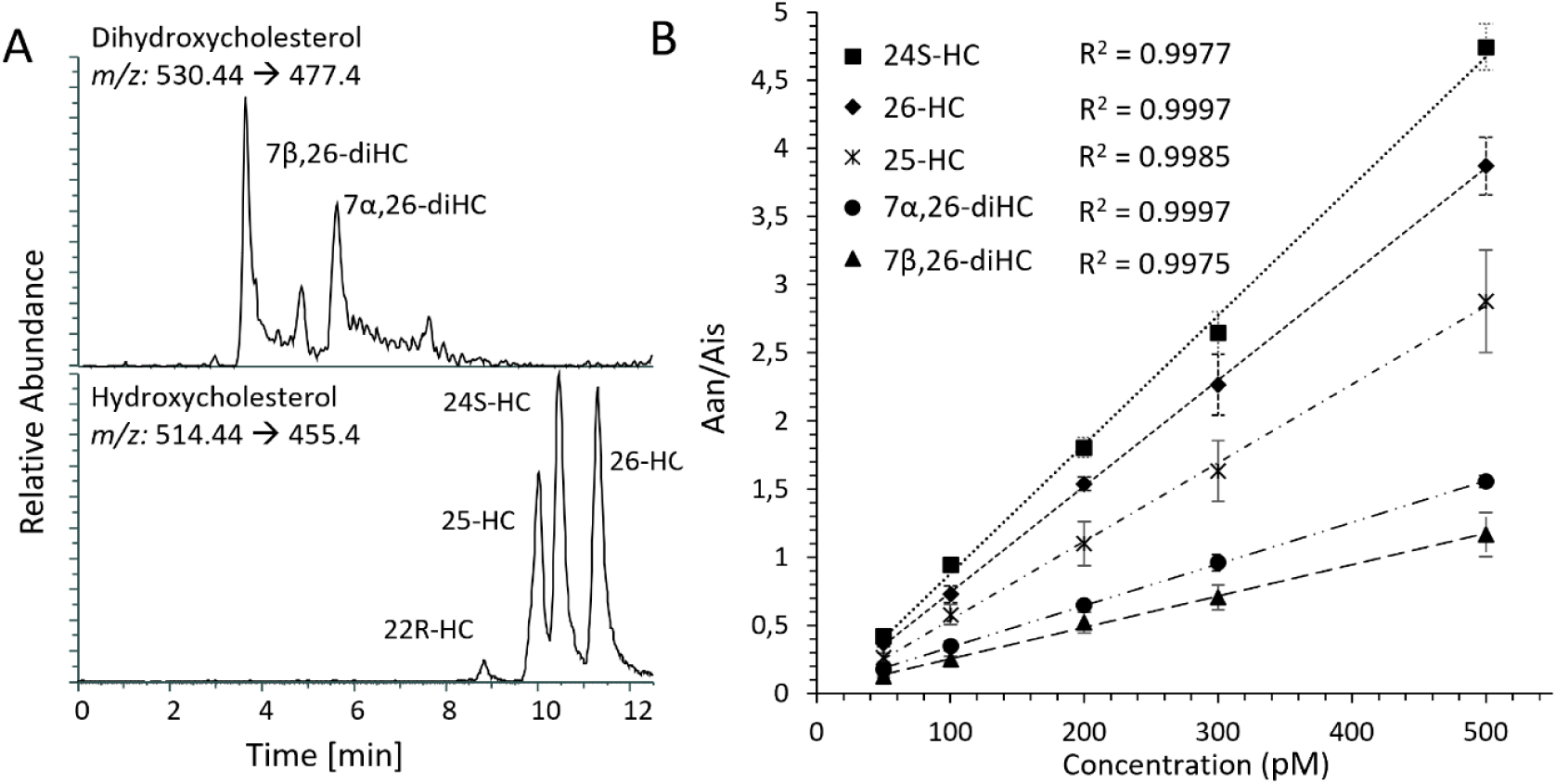
A) Chromatogram of oxysterols standard solution, with a concentration of 200 pM for each analyte, obtained during the process of validating the method. B) Mean calibration curves between days and operators for representative analytes. SD based on *n* = 4 is shown for each concentration.

Importantly, the relative error of less than 10% at both low- and high-level control samples (75 and 350 pM, 3 days, two operators) demonstrates the method’s accuracy and applicability for real-sample analysis, ensuring reliable results across a broad dynamic range. An absence of carryover (evaluated with blank injections after every third standard/sample) additionally highlights the suitability of the procedure for high-throughput applications, as analyte quantification in sequential samples will not be biased by residues from prior injections.

Overall, these findings confirm that the developed method meets internationally recognized quality assurance criteria (Eurachem guidelines^60^) and is thus appropriate for application in research-based analytical settings. Although matrix-spiked recovery was not feasible due to organoid heterogeneity, the Girard-T derivatization chemistry employed here has been extensively validated in complex biological contexts, including cells, exosomes, tissues, and plasma.^38,54,57,61–64^ This provides confidence in its suitability for organoid analysis. The complete set of validation data (including regression curves and supporting statistics) provides a comprehensive foundation for method reliability and transparency; see Supporting Information (SI-2, Tables S2–S4).

### Critical Assessment and Development of Normalization Procedure

When comparing oxysterol levels in gastruloids and organoids across various phenotypes and treatments,^65^ normalization can be required, i.e., determining the oxysterol concentration relative to protein content (fmol/μg). Our approach was to add *i*PrOH to single gastruloids/organoids, extract the oxysterols, and precipitate proteins. Measuring the total protein content (TPC) of a resolubilized protein pellet, while determining oxysterol levels in the separated *i*PrOH supernatant, would allow normalization and analysis using the same limited sample.

A commercial microBCA kit for low protein concentrations was first assessed, knowing that single organoid and gastruloid TPCs are in the low μg range. However, subtle differences in the matrix between calibration and sample solutions highly affect the sensitivity (i.e., slope) of the microBCA kit, jeopardizing its robustness and flexibility (Figure 4A).

**Figure 4:**
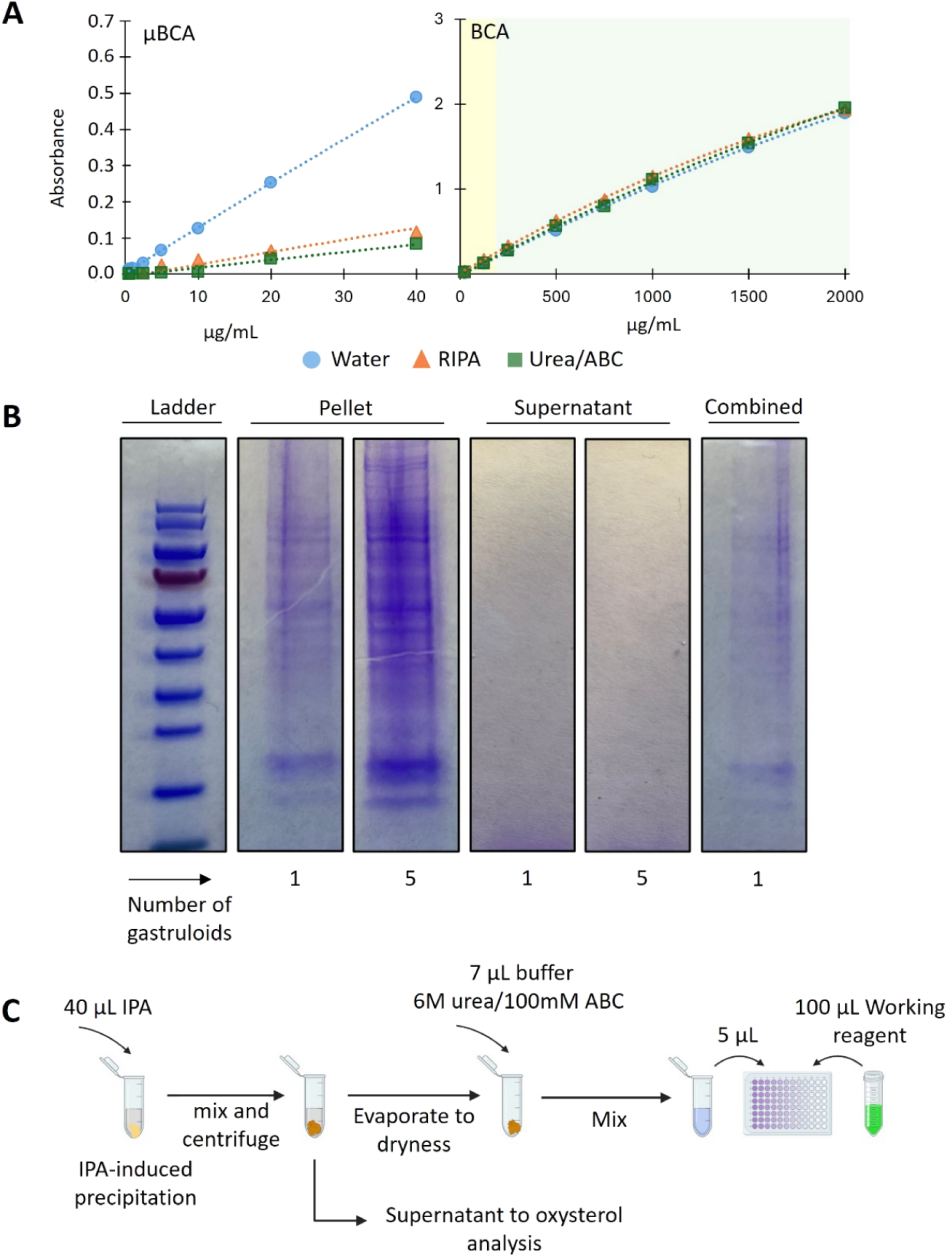
A) Calibration curve of BSA standard solutions for μBSA and conventional BCA assay analysis, using either water, RIPA buffer or Urea/ABC buffer as solvent. Background colors represent confidence in the calibration range: green indicates <20% relative error, yellow indicates >20% relative error. B) Results of SDS-PAGE analysis showing the efficiency of protein precipitation using various amounts of *i*PrOH for lipid extraction and various numbers of human gastruloids in the sample and a combined sample including both supernatant and pellet. C) Workflow of the normalization procedure.

Employing a conventional BCA kit for protein concentration measurements also warranted validation. We observed a relative error above 20% for quality control with protein concentrations lower than 200 μg/mL, suggesting that measuring concentrations below this level may lack accuracy (Figure 4A). Preliminary measurements with the standard protocol of the conventional BCA kit showed that protein concentrations in the single organoids and gastruloids are in the range of 30–100 μg/mL. We modified the kit protocol by lowering both the volume of solvent for dissolving the sample (from 25 to 5 μL) and the volume of the kit working reagent (from 200 to 100 μL), while simultaneously increasing the incubation time from 30 min to 2 h. These adjustments increased the final protein concentration after resuspension to approximately 250–700 μg/mL for organoids. Repeatable calibration curve slopes were obtained (*n* = 3, RSD < 4%) and QC measurements at 300 μg/mL (*n* = 3, RSD < 5%), supporting the robustness of the modified protocol.

Performing the lipid extraction and protein precipitation using 40 μL of *i*PrOH resulted in the absence of proteins in the supernatant (Figure 4B), with SDS-PAGE protein bands only visible in the protein pellet samples. Increasing the sample size from 1 to 5 gastruloids yielded stronger pellet bands, while still no protein bands appeared in the supernatant; the absence of protein bands in the supernatants indicates that virtually all proteins are precipitated. The visualized results were subsequently confirmed using the BCA kit: Protein concentrations above the LOD were found only in pellet samples, not in the respective supernatant.

Based on our evaluations, a protocol for *i*PrOH extraction of oxysterols that simultaneously allows for protein measurements of a single organoid was established (Figure 4C) and further investigations of biological samples were performed.

### Quantification of 26-HC in Single Human Liver Organoids (hscLO)

Applying our downscaled optimized sample preparation method, we mapped the presence of oxysterols in single hscLOs. Oxysterols were indeed detected with 26-HC present in sufficient amounts for quantification. Using human liver organoids originating from two cell lines (H1 and WTC-11), with 4–5 individual single hscLO samples from each cell line, 26-HC was quantified in the concentration range of 0.2–0.8 fmol/μg protein (Figure 5), demonstrating the feasibility of single organoid sterolomics. The data revealed heterogeneity between the hscLOs from the same cell line (RSD values of 45% and 73% for H1 and WTC-11 cell lines, respectively), which is comparable to other single organoid studies.^49^

**Figure 5:**
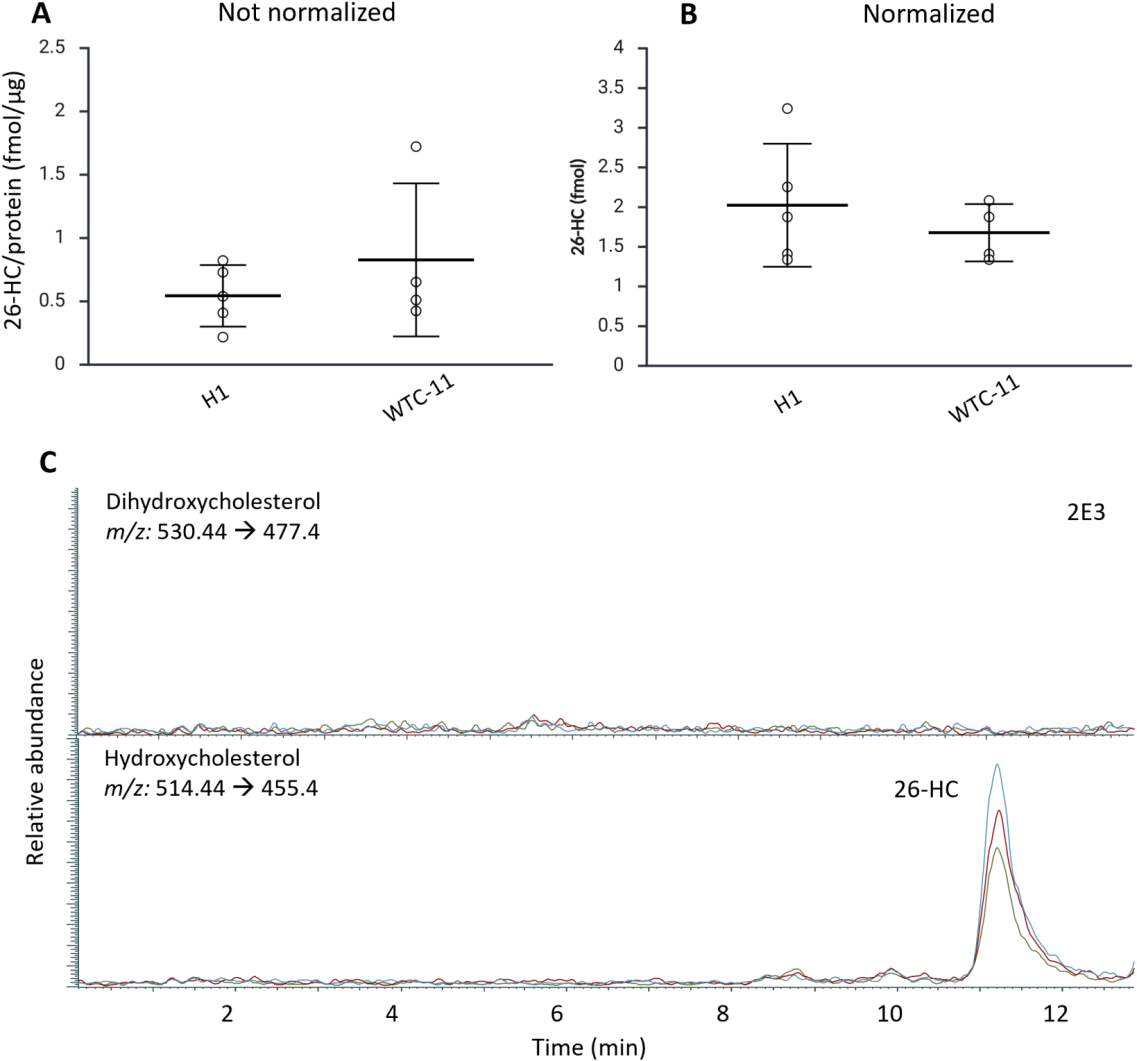
A) The amounts of 26-HC in hscLOs derived from two cell lines: H1 and WTC-11. B) The amounts of 26-HC in hscLOs derived from two different cell lines, H1 and WTC-11, normalized against protein content in the samples. C) Representative chromatogram of 26-HC in H1. Color lines represent replicates. No dihydroxycholesterols were detected in the organoids.

We found that the normalization of samples has a considerable effect on single hscLO quantification. When comparing protein content normalization of 26-HC (fmol/μg) versus no normalization (fmol/per single organoid) (Figure 5A and B), we found that the RSD in the normalized values for H1 increased from 38% to 45%, while for the WTC-11 cell line, RSD increased from 21% to 73% after normalization. Such considerable changes can reflect the heterogeneity of the hscLOs in terms of functionality and further suggest that normalization approaches should be carefully considered and ideally standardized to ensure comparable results between experimental settings and laboratories.

Other oxysterols, such as 24S-HC and 25-HC, were detected in a limited number of samples close to the method́s detection limits, showing that further increased sensitivity is needed to fully exploit the possibilities of single organoid sterolomics, for example, by downscaling the LC system to capillary format. However, our findings in single organoids correspond to earlier research on oxysterols secreted from human liver organoids, where we also found dominant levels of 26-HC,^38^ indicating higher CYP27A1 activity. To confirm that the oxysterols originated from the hscLOs and not the cell culture media used for cultivation, neat cell culture media was analyzed, and no traces of targeted oxysterols were detected in this media (SI-3, Figure S1). However, we and others have occasionally detected oxysterols in FBS-supplemented cell culture media previously, confirming the inevitable batch to batch content variability in FBS, underscoring the importance of cell media analysis when analyzing oxysterols in cells and organoids. Additionally, hscLOs prepared without the use of cholesterol oxidase also showed no peaks for targeted oxysterols, confirming that the detected oxysterols do not originate from 3-oxo-4-ene sterols (SI-3, Figure S1).

### 26-HC, 24S-HC, and an Unidentified Oxysterol Quantified in Single Gastruloids

Next, the method was applied to analyze oxysterols in single human gastruloids. Oxysterols are known as regulators in Hedgehog and Wnt signaling,^66^ which are key pathways in early development. The results of preliminary proteomic analysis that shows the presence of proteins related to Wnt and Hedgehog pathway activity (Smoothened and GLI) support that gastruloids recapitulate critical molecular events of early human development. By applying our method on single gastruloids, we could detect and quantify 24S-HC and 26-HC (Figure 6), in addition to semiquantification of an unidentified dihydroxycholesterol in all human gastruloid samples (quantification based on a calibration curve for the dihydroxycholesterol 7β26-diHC). The capability to detect developmental regulators such as oxysterols in these *in vitro* human models underscores the power of this approach, revealing pathway activity that to our knowledge has not been previously demonstrated in model systems of this type. To again confirm that the oxysterols were produced in the human gastruloids and did not originate from the cell culture media, neat cell culture media was analyzed for oxysterols; no traces of oxysterols were identified (SI-3, Figure S1). In addition, exclusion of naturally occurring 3-keto sterols was performed by performing sample preparation with and without cholesterol oxidase (SI-3, Figure S1). High-resolution fragmentation spectra (MS/MS) confirm the identity of a dihydroxycholesterol (Figure 6D), together with high-resolution (HR) MS data (SI-4, Figure S2).

**Figure 6:**
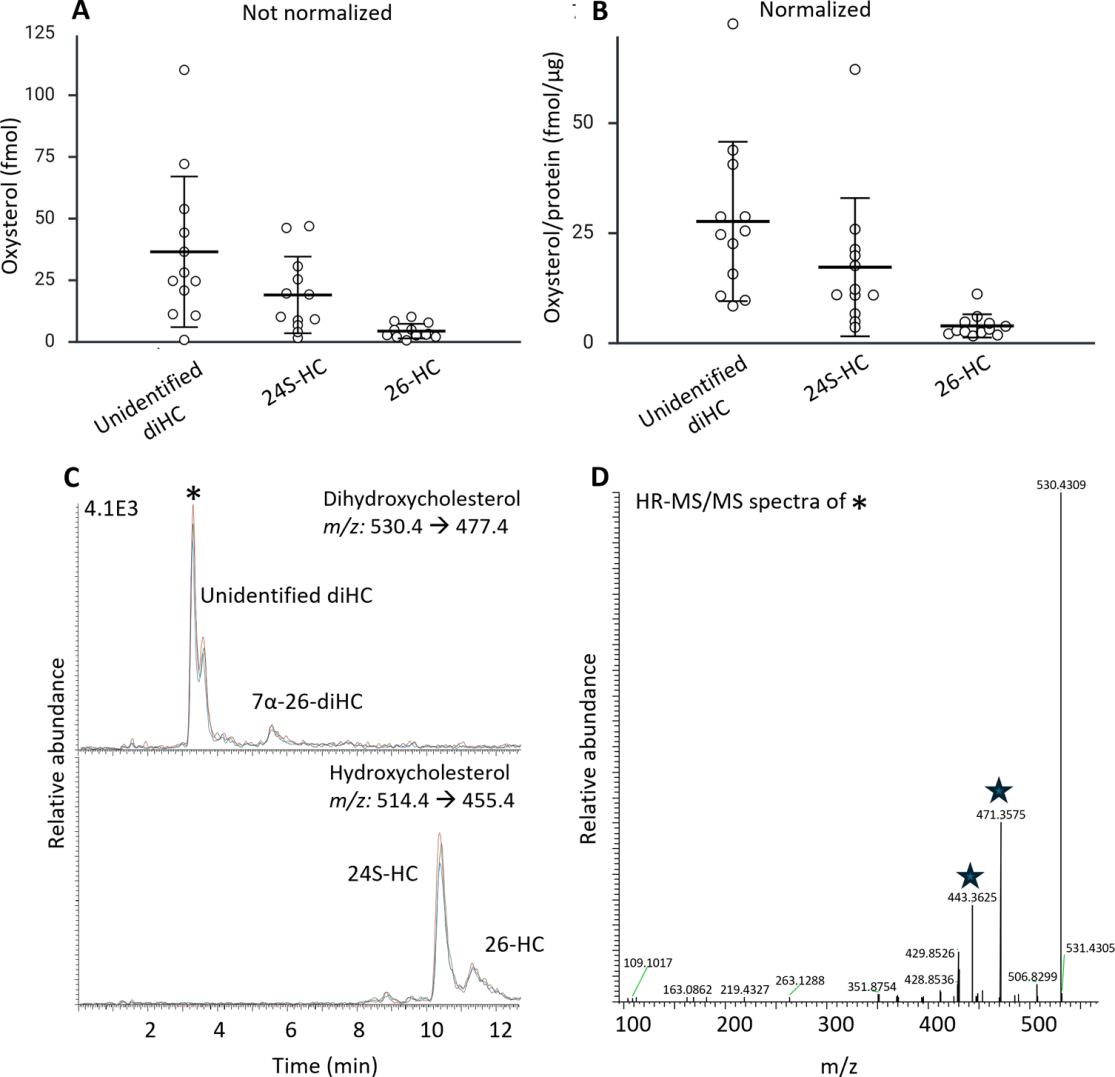
A) Amounts of 24S-HC, 26-HC, and an unidentified diHC in human gastruloids. B) Amounts of the same oxysterols normalized against protein content. C) Representative chromatogram of hydroxycholesterols and dihydroxycholesterol detected in human gastruloids. Color lines represent replicates normalized to the respective internal standard. D) HR-MS/MS spectra of the unidentified dihydroxycholesterol marked with * in 6C. Expected fragments of *m*/*z* 471 and 443 are marked with a star. For MS details, see SI-4.

We could also detect 7α26-diHC but below the limit of quantification. In attempts to identify the most abundant (but unknown) dihydroxycholesterol, we compared the retention times to a set of relevant standards, but the mismatch allowed us to exclude these as candidates (Figure 7).

**Figure 7:**
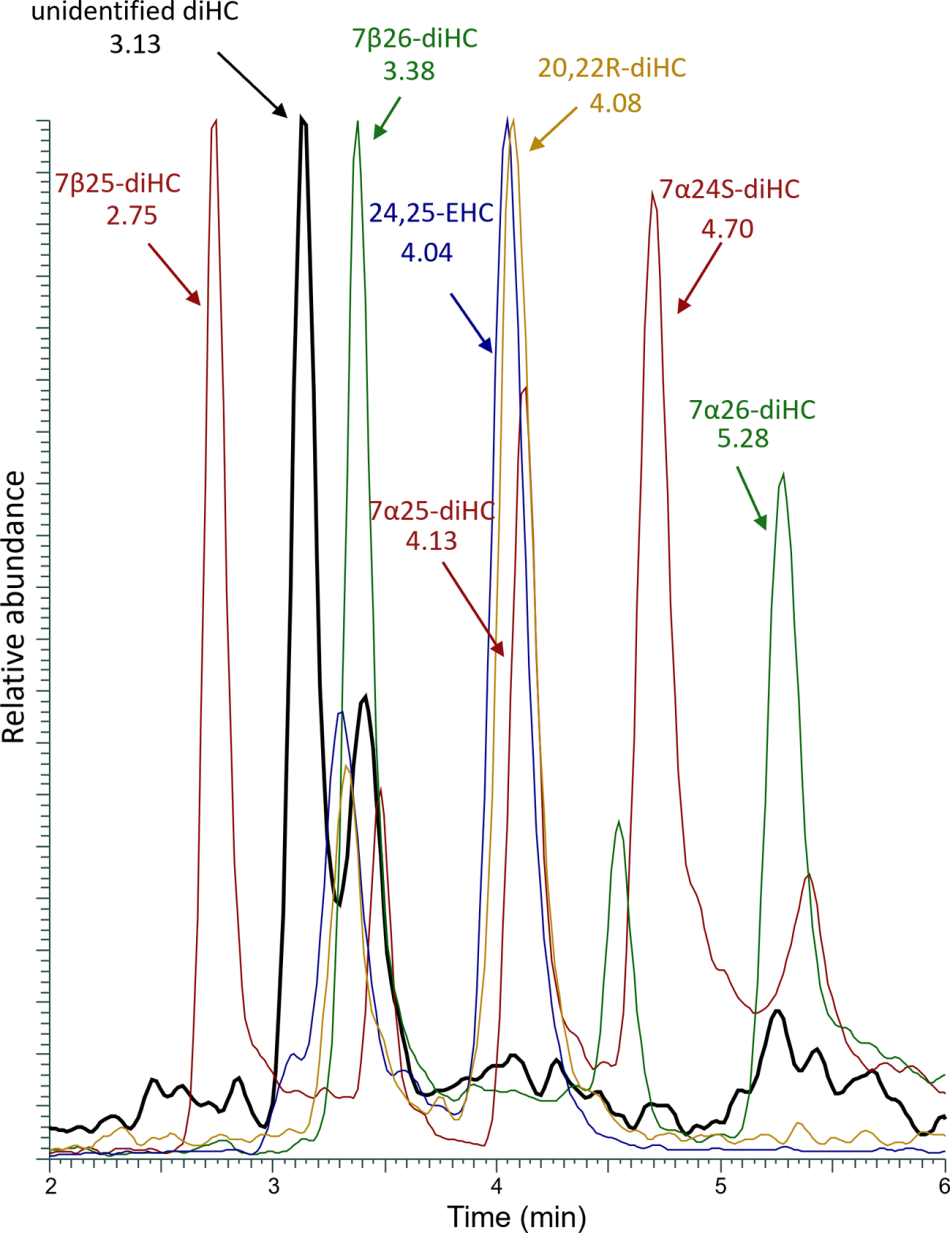
Overlay of chromatogram comparing retention times of standard dihydroxycholesterols and the unidentified compound detected in gastruloid samples.

The presence of 24S-HC was unexpected, as this hydroxycholesterol is mainly associated with transport through the blood–brain barrier^67,68^ and the formation of cancerous cells.^69,70^ To exclude the possibility of a misidentification with other oxysterols with identical masses and fragment patterns, a comparison based on the retention time match was performed (Figure 8), strengthening the identification of 24S-HC.

**Figure 8:**
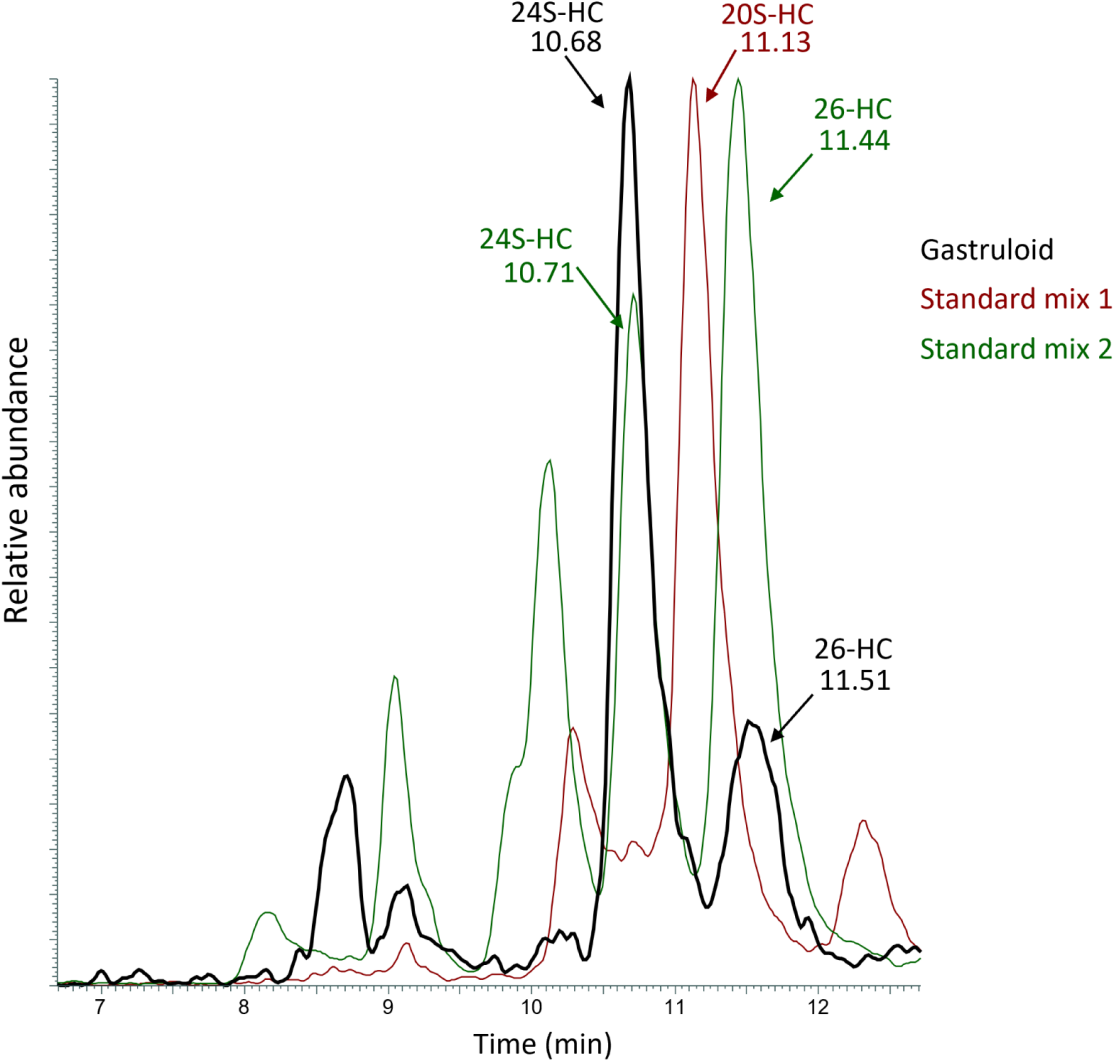
Overlay of the chromatogram comparing retention times of standard hydroxycholesterols and the potential 24S-HC detected in gastruloid samples.

The concentration of 26-HC was found at the lowest level of the three quantifiable compounds (2–11 fmol/μg protein, RSD = 70%) followed by 24S-HC (4 – 62 fmol/μg protein, RSD = 92%) and the unidentified dihydroxycholesterol (10–72 fmol/μg protein, when applying the calibration curve for 7β26-diHC, RSD = 66%). The excessive standard deviations and differences between the individual human gastruloids confirm significant heterogeneity, underlining the importance of investigating single human gastruloids rather than pooled samples.

## Conclusions

We report a miniaturized sample preparation protocol for oxysterol derivatization prior to LC–MS analysis, achieving a 10-fold reduction in sample volume without compromising sensitivity or method performance. Optimization through experimental design enabled a systematic evaluation of reagent contributions to the reaction yield under limited sample conditions, revealing the opportunity to significantly reduce solvent volumes and hence sample dilution. The validated method shows, e.g., satisfactory repeatability, linearity, and accuracy. Isopropanol was employed for both oxysterol extraction and protein precipitation, allowing for same-sample analysis and normalization. Our final method enabled mapping of oxysterols and quantification of 26-HC in a single hscLOs, and both 24S-HC and 26-HC in single human gastruloids, in addition to an unknown dihydroxycholesterol, the role of which—if any—in human gastruloids and embryogenesis remains to be determined. The observed heterogeneity among individual hscLOs and human gastruloids supports the need for single-sample analysis. This study is a first step in single-sample sterolomics for models of liver function and early embryogenesis, and further steps to enhance sensitivity (e.g., separation column downscaling) and a comprehensive single-organoid/gastruloid lipidomics profiling will be studied next.

## Supplementary Material



## Notes

Provided in a separate file and contain comprehensive mass spectrometric (MS) parameters, evaluation and validation metrics, as well as confirmatory data on oxysterol abundance in liver organoids and human gastruloids (PDF)
