# Supplementary material for: A Validated Mass Spectrometry Platform for Oxysterol Analysis of Single Human Gastruloids and Liver Organoids
Source: Anal Chem. 2026 Feb 24;98(9):6792–804. doi: 10.1021/acs.analchem.5c07140 (PMC12980495; doi:10.1021/acs.analchem.5c07140)
Supplement: Supplementary file 1 [file ac5c07140_si_001.pdf]

Supporting Information for:

A Validated Mass Spectrometry Platform for Oxysterol Analysis of Single Human Gastruloids and Liver Organoids

Kristina Sæterdal Kømurcu[a,b,†], Malgorzata Elzbieta Zawadzka[a,b,†], Igor Meszka[b], Aleksandra Aizenshtadt[b,d], Helena Hrušková[a], Lydia Emilie Aakervik[a], James L Thorne[c], Steven Ray Wilson[a,b], Stefan Johannes Karl Krauss[b,d], and Hanne Røberg-Larsen[a,b]*

[a]Department of Chemistry, University of Oslo, P.O. Box 1033 Blindern, 0315 Oslo, Norway,
[b]Hybrid Technology Hub - Centre of Excellence, Institute of Basic Medical Sciences, University of Oslo, P.O. Box 1110 Blindern, 0317 Oslo, Norway
[c]School of Food Science and Nutrition, University of Leeds, Leeds LS2 9JT, United Kingdom
[d]Department of Immunology and Transfusion Medicine, Oslo University Hospital, Oslo, Norway.

[†]Shared first authorship

*Corresponding author: E-mail: hannero@kjemi.uio.no. Telephone: +47 900 20 101

SI-1 MS parameters

The TSQ Quantiva triple quadrupole mass spectrometer was operated in positive mode with a voltage of +3500V, collision gas pressure of 1 mTorr, sheat gas pressure of 60 Arb, and an auxiliary gas flow of 10 Arb. The capillary temperature was set to 380 °C, and the vaporizer temperature was 300°C. All analytes were monitored in selected reaction monitoring mode (SRM), see **Table S1** for more information.

Table S1. SRM-transitions and collision energy for all targeted analytes, both quantifier and qualifier.

Analytes	Parent m/z	Collision energy	Quantifier m/z	Qualifier m/z
7α,25-diHC<br>7α,26-diHC<br>7β,26-diHC<br>7β,25-diHC<br>7α,24S-diHC	530.4	34/37	471.4	443.4
7β,26-diHC-d_6	536.4	34/37	477.4	449.4
22R-HC<br>24S-HC<br>25-HC<br>26-HC	514.4	33/35	455.4	427.4
25-HC-d_6<br>27-HC-d_6	520.4	34/37	461.4	433.4
Cholesterol autoxidation monitoring	517.4	34	458.4	

SI-2 Validation

Table S2. Data for method validation, including calibration curve equation, R^2-values and results of ANOVA analysis for the intra- and inter-day repeatability at three different concentration levels.

**Analyte**	**Range**	**LOD/LOQ**	**y=ax+b (R^2)**	**Precision LLOQ (50 pM)**		**Precision MLOQ (200 pM)**		**Precision HLQ (500 pM)**	
	(pM)	(pM)		Intra-day (n=6) RSD	Inter-day (n=3) Anova	Intra-day (n=6) RSD	Inter-day (n=3) Anova	Intra-day (n=6) RSD	Inter-day (n=3) Anova
25-HC	50-500	10/33	0.0058x-0.0338 (0.999)	7	12	9	17	11	17
24S-HC	50-500	15/50	0.0095x-0.0713 (0.998)	9	9	8	9	12	13
26-HC	25-500	7/24	0.0078x+0.0039 (0.999)	8	9	5	5	9	10
7β26-diHC	50-500	15/51	0.0031x+0.0331 (0.999)	15	15	9	9	8	8
7α26-diHC	100-500	29/99	0.0023x+0.0221 (0.998)	19	19	12	15	13	15

Analogically the repeatability between the operators was tested using Student t-test and it shows that the precision of the method is not dependent on the operator for any of the analytes.
The *p-values* for all measurements are above 0.05 and are presented in **Table S3**.

Table S3. P-values for two-tailed t-test.

	**25-HC**	**24S-HC**	**26-HC**	**7β26-diHC**	**7α26-diHC**
50 - LQ	0.02	0.28	0.85	0.17	0.71
200 - MQ	0.99	0.74	0.85	0.26	0.10
500 - HQ	0.79	0.83	0.56	0.81	0.08

The concentration of control samples was tested on to levels, 75 pm and 350 pM for each day and operator. As presented in **Table S4**, the average relative error for nearly all measurements was within 10% (except for 7β26-diHC and 7α26-diHC at the lower concentration).

Table S4. Average relative error.

**Analyte**	**nominal concentration (pM)**	**average conc (pM)**	**average relative error (%)**
25-HC	75	80	8
	350	348	3
24S-HC	75	80	6
	350	344	4
26-HC	75	78	6
	350	359	7
7β26-diHC	75	77	12
	350	380	8
7α26-diHC	75	62	26
	350	381	7

SI-3 Confirmation of oxysterol abundance in human liver organoids and human gastruloids

To verify the origin of detected oxysterols, analysis of cell culture media with supplements used to culturing the single human organoids and gastruloids were analyzed. Neither of the cell culture media contained detectable amounts of the oxysterols (**Figure S1**).

In addition, to confirm oxysterol identity and exclude the 3-oxo-delta-4 ketones (that already possess the 3-positioned ketogroup that in this method is introduced by step one in the derivatization reaction) sample preparation without the cholesterol oxidase was performed. Both cell culture media, gastruloids and organoids were analyzed without cholesterol oxidase. No oxysterols were detected in neither cell culture media or in the organoids or gastruloids. This confirms that all the detected oxysterols, come exclusively from gastruloids and organoids, and can be identified as specific hydroxy- and dihydroxycholesterols.

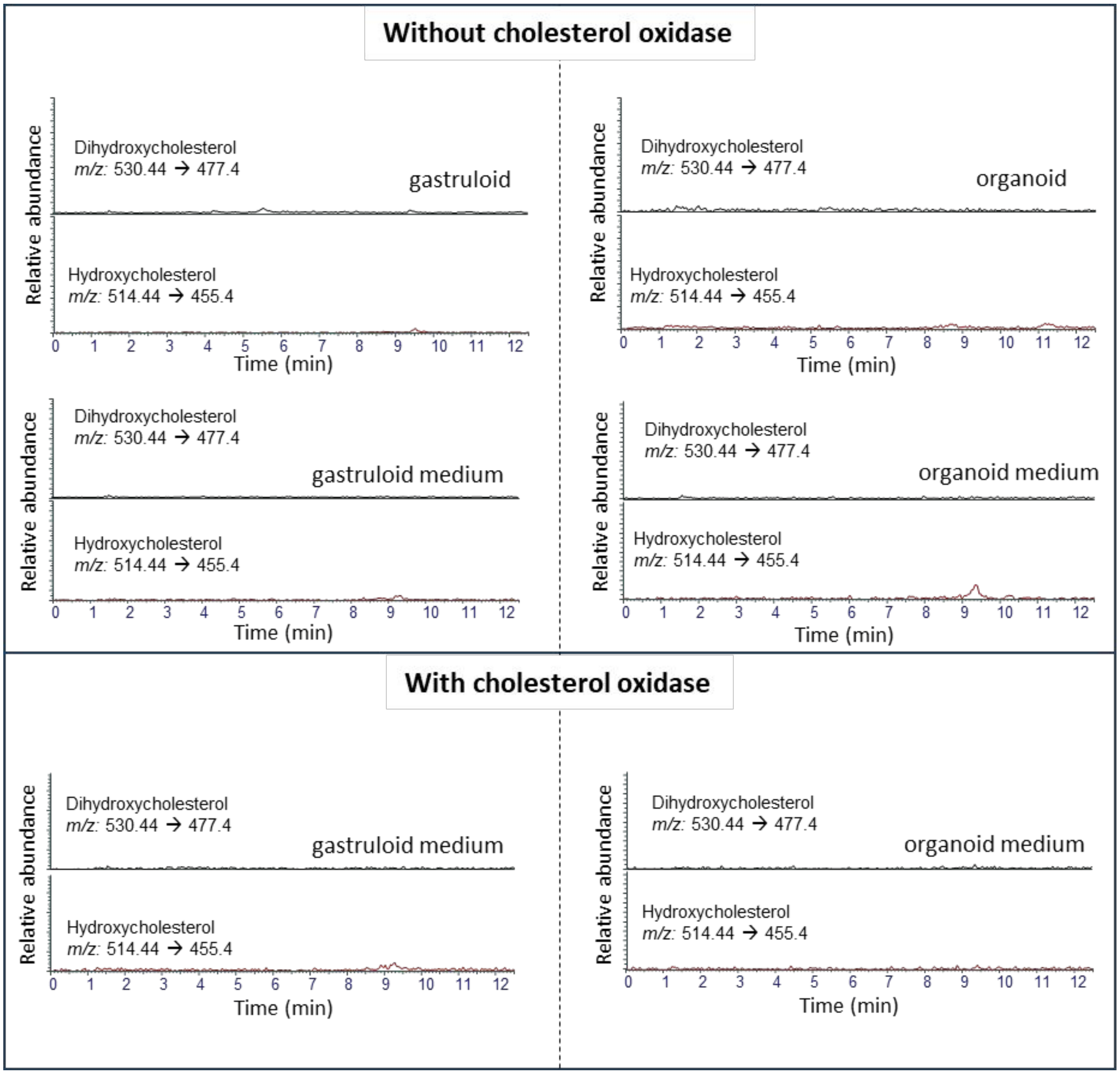

Figure S1. Chromatograms of samples analyzed with and without cholesterol oxidase, to confirm origin of detected oxysterols. Samples include a human gastruloid, gastruloid cell medium, human liver organoid and liver organoid cell medium. Results show that no oxysterols are detected besides the ones coming directly from gastruloids and organoids and derivatized during sample preparation.

SI-4 High resolution MS of gastruloid samples

Gastruloid sample was analyzed using the LC-setup described in the main text, coupled to a high resolution MS QExactive Orbitrap (Thermo Fisher Scientific, Waltman, MS, USA) equipped with an electrospray ionization (ESI) source operating in positive ion mode.

In both full scan and PRM the capillary voltage was 3.5 kV, the drying gas temperature 300 °C, and the capillary temperature 380 °C. AGC target was set to 1e5, the maximum injection time to 100 ms and S-lens value was set to 80. Sheat gas flow rate was 56 a.u., the auxiliary gas flow rate was 16 a.u. and the sweep gas flow rate 3 a.u. Both methods duration was 14.5 min. In full scan the mass range was set to *m/z* 380 – 580 with resolution of 140 000. PRM analysis was carried with the resolution of 17 500, the isolation window was set to 1.5 *m/z* and the collision energy (NCE) to 35. An inclusion list containing masses: 514.44 *m/z*; 520,47 *m/z*; 530,43 *m/z*; 536.47 *m/z* was applied, with the normalized collision energy (NCE) set to 36. Method duration was 14.5 min. Full MS chromatograms of single gastruloid and mass spectra of peaks of interest are shown in **Figure S2.**

Extracted ion chromatograms are performed with a mass tolerance of 5 ppm.

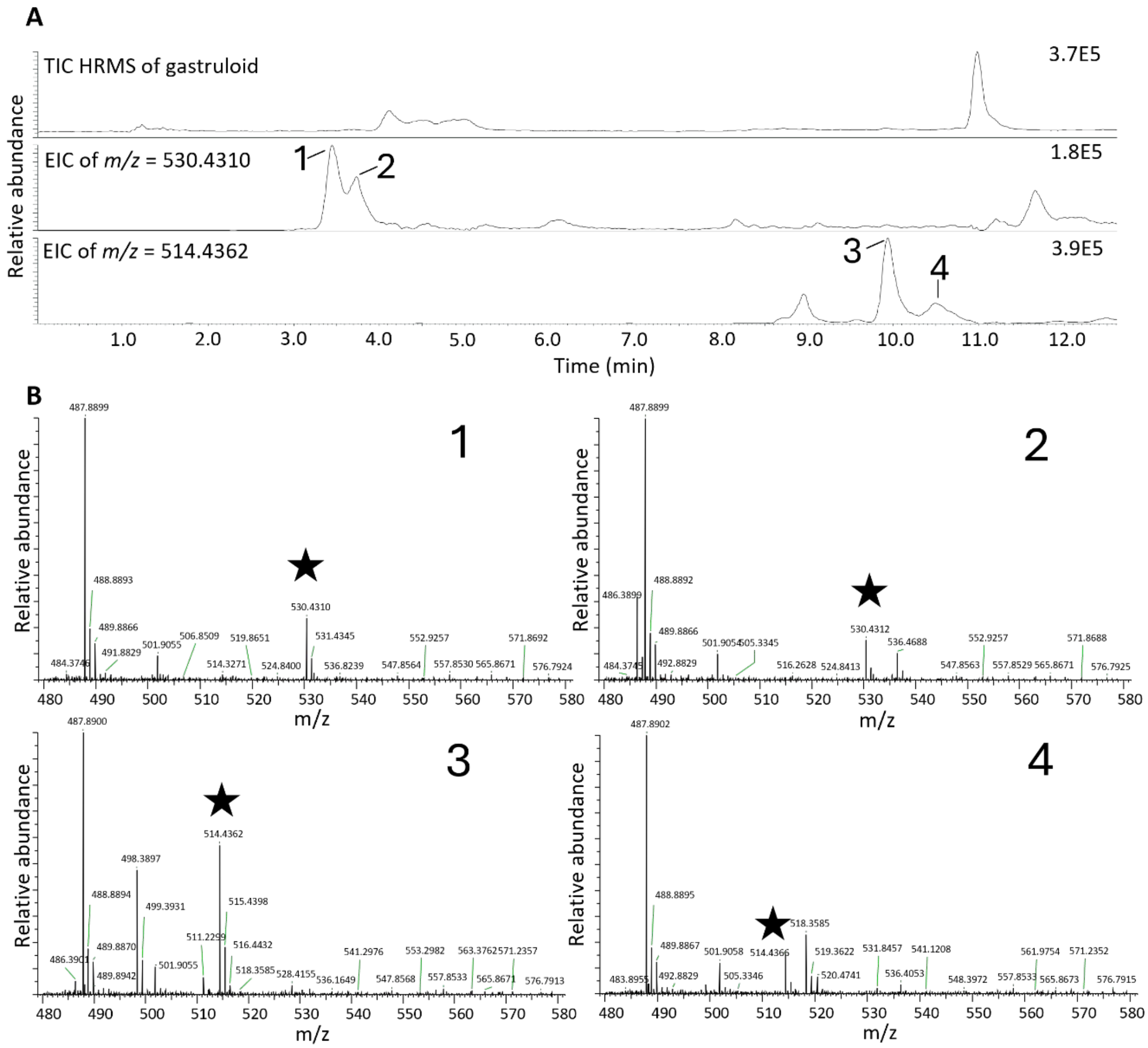

Figure S2. A) Full HRMS scan of a single gastruloid with TIC and extracted ion chromatogram (EIC) of *m/z* 530.4310 (dihydroxycholesterols) and 514.4352 (hydroxycholesterols), respectively. Numbers 1-4 mark the target peaks. B) Mass spectra of peaks 1-4 marked in A. Targeted *m/z* peaks are marked with a star.
